# Vancomycin-resistant *Enterococcus faecalis* in Serbia

**DOI:** 10.3201/eid1001.020790

**Published:** 2004-01

**Authors:** Branka Stošović, Srdjan Stepanović, Susan Donabedian, Tanja Tošić, Milica Jovanović

**Affiliations:** * Institute of Infectious and Tropical Diseases “Dr Kosta Todorović,” Belgrade, Serbia; † University of Belgrade School of Medicine, Belgrade, Serbia; ‡ William Beaumont Hospital, Royal Oak, Michigan, USA

**Keywords:** antibacterial drug resistance, *Enterococcus faecalis*, vancomycin resistance

**To the Editor:** First isolated in France (*1*), vancomycin-resistant enterococci (VRE) have become pathogens of major importance, particularly in the United States (*2*). Infections due to VRE are still uncommon in most European countries (*3*). We report the first isolation of high-level vancomycin-resistant *Enterococcus faecalis* in Serbia.

 A 55-year-old woman was admitted to the Clinic for Cardiovascular Diseases, Belgrade, on April 1, 2002, for aortobifemoral bypass surgery. Three weeks after she was admitted to the hospital, an infection developed in the surgical wound and treatment with sulfamethoxazole-trimethoprim (160/800 mg q 12 h) was empirically introduced. Bacteriologic analysis of the wound swab sample showed a methicillin-resistant strain of *Staphylococcus aureus*, a multiresistant strain of *Acinetobacter* sp., a commonly susceptible strain of *Enterococcus* sp., and a VRE strain. According to the results of susceptibility testing, imipenem (1 g q 6 h) was added to the patient’s treatment protocol. VRE were not isolated from subsequent wound samples or any other sample submitted for microbiologic analysis. The patient was discharged at the end of the 14-day treatment period.

 The isolate was identified as *E. faecalis* by biochemical characterization, as recommended by Facklam and Collins (*4*) and confirmed by API 20 Strep (bioMérieux, Marcy-l’Etoile, France). Susceptibility testing, performed by the disk diffusion method, showed that the isolate was resistant to vancomycin, teicoplanin, gentamicin, streptomycin, tetracycline, and ciprofloxacin, while susceptible to ampicillin, amoxicillin, amoxicillin and clavulanic acid, and imipenem. Resistance to vancomycin, teicoplanin, gentamicin, and streptomycin was confirmed by the broth dilution method, according to the National Committee for Clinical Laboratory Standards (NCCLS) recommendations (*5*). The obtained MICs were 256 μg/mL for vancomycin, 64 μg/mL for teicoplanin, >4,000 μg/mL for gentamicin, and >2,000 μg/mL for streptomycin. This phenotype, with high-level resistance to vancomycin and teicoplanin, is typical for the *vanA* genotype (*2*). The strain was subsequently genotyped by pulsed-field gel electrophoresis, using previously described methods (*6*). The presence of the *vanA* gene was confirmed by polymerase chain reaction assay, according to a previously described procedure (*7*). *E. faecium* EF228 was used as the positive control.

 The enterococci are among the most frequent causes of nosocomial infections, particularly in intensive care units, and present a major therapeutic challenge (*2*). While the emergence of VRE strains in the United States is probably associated with extensive use of vancomycin, the occurrence of VRE in Europe is possibly due to application of avoparcin (glycopeptide analog) as a growth promoter in animal husbandry (*3*). However, avoparcin has not been used in Serbia, and vancomycin application has been restricted to hospitalized patients and quite limited due to its high cost. Thus, emergence of VRE strains in Serbia has not been likely.

The origin of this VRE isolate is unknown: the strain may have been imported or may have originated from the hospital environment. The first prospective pan-European VRE surveillance study (January–April 1997) showed VanA-VRE strains in only eight European countries, with isolates numbering from one to four per country (*3*). No epidemiologic relations were established among the VanA isolates, and only 2 out of 18 isolates (11%) were identified as *E. faecalis* (*3*). Since our patient-case had no history of travel outside Serbia, we assumed that the VRE isolate originated from the hospital environment. However, a study investigating the occurrence of VRE strains in Belgrade, the capital of Serbia, detected no such isolates in five different hospitals (*8*). Although the study did not analyze samples from the Clinic for Cardiovascular Diseases, it did include samples from the Clinic for General Surgery, which is located within the same building. The susceptibility of 191 isolates of enterococci to vancomycin was tested by agar dilution method according to NCCLS recommendations. Of the 191 isolates, 159 were classified as susceptible and 32 as intermediately susceptible.

 This report of the first isolation of VRE in Serbia, as well as the previously shown presence of enterococci displaying intermediary susceptibility to vancomycin, provides the rationale for future active screening for VRE in hospital environments in the region.
